# Improved EfficientNet for corn disease identification

**DOI:** 10.3389/fpls.2023.1224385

**Published:** 2023-09-11

**Authors:** Jitong Cai, Renyong Pan, Jianwu Lin, Jiaming Liu, Licai Zhang, Xingtian Wen, Xiaoyulong Chen, Xin Zhang

**Affiliations:** ^1^ College of Big Data and Information Engineering, Guizhou University, Guiyang, China; ^2^ Guizhou-Europe Environmental Biotechnology and Agricultural Informatics Oversea Innovation Center in Guizhou University, Guizhou Provincial Science and Technology Department, Guiyang, China; ^3^ International Jointed Institute of Plant Microbial Ecology and Resource Management in Guizhou University, Ministry of Agriculture, China Association of Agricultural Science Societies, Guiyang, China; ^4^ College of Life Sciences, Guizhou University, Guiyang, China

**Keywords:** Convolutional Neural Network, corn leaf disease, real scene, lightweight model, fully-convolution-based coordinate attention

## Abstract

**Introduction:**

Corn is one of the world's essential crops, and the presence of corn diseases significantly affects both the yield and quality of corn. Accurate identification of corn diseases in real time is crucial to increasing crop yield and improving farmers' income. However, in real-world environments, the complexity of the background, irregularity of the disease region, large intraclass variation, and small interclass variation make it difficult for most convolutional neural network models to achieve disease recognition under such conditions. Additionally, the low accuracy of existing lightweight models forces farmers to compromise between accuracy and real-time.

**Methods:**

To address these challenges, we propose FCA-EfficientNet. Building upon EfficientNet, the fully-convolution-based coordinate attention module allows the network to acquire spatial information through convolutional structures. This enhances the network's ability to focus on disease regions while mitigating interference from complex backgrounds. Furthermore, the adaptive fusion module is employed to fuse image information from different scales, reducing interference from the background in disease recognition. Finally, through multiple experiments, we have determined the network structure that achieves optimal performance.

**Results:**

Compared to other widely used deep learning models, this proposed model exhibits outstanding performance in terms of accuracy, precision, recall, and F1 score. Furthermore, the model has a parameter count of 3.44M and Flops of 339.74M, which is lower than most lightweight network models. We designed and implemented a corn disease recognition application and deployed the model on an Android device with an average recognition speed of 92.88ms, which meets the user's needs.

**Discussion:**

Overall, our model can accurately identify corn diseases in realistic environments, contributing to timely and effective disease prevention and control.

## 1 Introduction

Corn is a globally important crop with high nutritional value and significant economic importance for farmers (Rosas-Castor et al., 2014). Corn is affected by a variety of unfavorable factors during the planting process, among which disease is the main disaster that affects corn yield, leading to a significant reduction in the yield of diseased corn and causing economic losses to farmers. Therefore, timely detection of corn diseases is crucial. However, traditional methods of diagnosing corn diseases, which require diagnosticians to physically enter the field and rely on preliminary diagnosis or quantitative analysis to determine the type of disease, are both time-consuming and labor-intensive. Agricultural experts and technicians cannot reach the field in time, while most farmers lack the necessary knowledge to accurately diagnose diseases, resulting in time, cost, and inefficiency in problem solving. In the field of plant disease recognition, existing research suggests two main approaches: traditional machine learning and deep learning (DL).

Traditional image processing methods has improved the efficiency of corn disease prevention and control to some extent (Guruprasad, 2020; O’Mahony et al., 2020). Kai et al. (2011) segmented the lesions based on the texture characteristics of corn diseases using the YCbCr color space technique, extracted the lesion texture features using the co-occurrence matrix spatial gray-level layer, and classified corn diseases using a BP neural network, achieving an accuracy rate of 98%. Aravind et al. (2018) processed the image to obtain a feature bag and texture features based on statistical histograms, and used a multi-class support vector machine to classify the diseases based on the obtained features, achieving an average best accuracy rate of 83.7%.

Recognizing crop diseases using machine learning algorithms requires features to be designed manually, which is time-consuming and laborious. In contrast, DL models are able to autonomously learn information about image target features, and are therefore widely used in the field of image recognition. Praveen et al. (2022) using the FastAI technology with ResNet-32 to diagnose ductal carcinoma, The experimental results show that 93.60% recognition accuracy was achieved on IDC dataset. Srinivasu et al. (2022) proposed an RNN model integrating GRU and LSTM with auxiliary memory components and designed one, for predicting type 2 diabetes, and the experimental results showed that the model achieved a correct recognition rate of 81.8%. For plant disease recognition, Nigam et al. (2023) used fine-tuned EfficientNet-B4 model for recognizing wheat disease and the experimental results showed that 99.35% recognition accuracy was obtained on their collected dataset. Al-Gaashani et al. (2023) based on the ResNet50 architecture, combined with kernel attention mechanism proposed SANET for recognizing rice disease, and the experimental results showed that 98.71% recognition accuracy was obtained on the publicly available rice disease dataset.

In the above study, the images of crop disease datasets usually have a simple background collected in the laboratory, while in the real environment, it is difficult to distinguish the differences between disease features with complex backgrounds to achieve the accuracy of specific disease identification in the previous model. The use of max pooling can highlight the most significant features, help filter some noise, and reduce the interference from complex backgrounds. Sun et al. (2020) proposed a novel max pooling method for CNN trained by noisy samples, and the classification accuracy of CNN with this method for noisy images is much higher than that of traditional pooling methods. Lin et al. (2022b) proposed CAMFFNet for tobacco disease identification using multiple feature fusion module, which introduced additional max pooling branches for feature extraction at different locations of the structure. The results showed that the recognition accuracy of the test set was 89.71%.At the same time, for complex background images, the attention mechanism can make the model focus on the disease region in a targeted manner, improve the ability of the model to learn disease features, and reduce the influence of the model by noise to improve the accuracy of the model. Hu et al. (2023) proposed a Class-Attention-based Lesion Proposal Convolutional Neural Network to locate disease objects of complex backgrounds, and the experimental results showed that the recognition accuracy was 92.56% on a self-constructed field strawberry disease dataset. Stephen et al. (2023) designed a self-attention-based ResNet architecture for rice disease classification, and the experimental results showed that the accuracy of recognition of four types of rice diseases was 98.54%.

The above studies have well demonstrated the superiority of CNN for plant disease identification. However, the above models are heavy-weight and require large computational resources, which are not suitable for efficient deployment on mobile devices for timely disease identification. Therefore, there is a need to design lightweight networks that can be deployed on mobile devices. Vishnoi et al. (2022) proposed a new CNN model using only fewer convolutional layers to reduce the computational burden, and the experimental results showed a 98% classification accuracy for four classes of apple diseases. Lin et al. (2022a) proposed a lightweight CNN model called GrapeNet for recognizing specific grape diseases at different symptom stages, and the experimental results showed that the recognition accuracy for seven classes of grape diseases was 97.85%. Chen et al. (2022) proposed a lightweight CNN model, DFCANet, for recognizing corn diseases in real environments, and the experimental results showed that the classification accuracy for six classes of corn diseases reached 98.47%.

Identification of corn leaf diseases in field environments faces several difficulties, such as complex background disturbances, variability and irregularity of lesion areas. In addition, traditional CNN models with a large number of parameters in crop identification tasks require more computational resources, cannot be deployed on mobile devices and are difficult to scale widely.

To solve the above problems, we focus on corn diseases and propose a lightweight CNN model with high recognition accuracy, FCA-EfficientNet. on top of EfficientNet, we use adaptive fusion (AF) module for shallow feature extraction, fully-convolution-based coordinate attention (FCA) module to focus on disease areas in complex backgrounds, better normalization methods and activation functions to enhance the recognition of the network speed, and removing the network redundant structure of the corn disease dataset to make the network easier to deploy. This study explores the accurate recognition of corn disease images and provides new insights for plant disease recognition research. The main contributions of this study are as follows:

- Since the field environment often has complex background noise, in order to reduce the interference of background on the network recognition results, we use pooling layer to reduce the background interference, and through the AF module, fuse the features of pooling layer and convolutional layer, so that the network can better extract shallow information and improve the recognition accuracy.
- To better focus the network on the lesion area while reducing the interference of background noise, we propose the FCA module, which uses a convolutional structure to extract spatial information, allowing the network to better extract disease areas and reduce the recognition difficulty.
- In order to make the network have better generalization ability, we use more advanced normalization method with activation function to improve the robustness of the network.
- To make the model easy to deploy due to performance limitations of mobile devices, we removed the redundant structure of the model to make the model have faster inference speed and smaller number of parameters.

The paper generally consists of several sections. The “Introduction” section provides a brief overview of the research field, motivation, and objectives. The “Materials and Methods” section describes the source of the dataset and the methods used for data processing. The “Proposed method” section provides an explanation of the technical details adopted in this paper. The next section presents the “Experimental results and analysis”. The “Conclusions” section provides conclusions and outlines future research directions. Finally, the last section includes the references cited in this paper.

## 2 Materials and methods

### 2.1 Image acquisition

**Figure 1:**
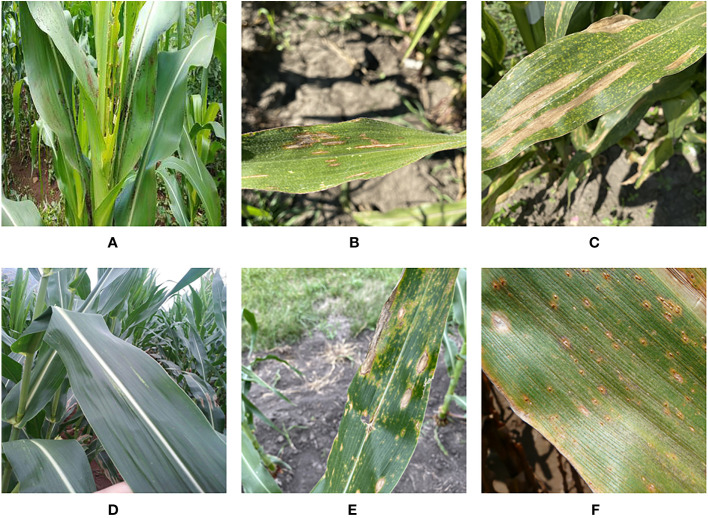
Example of corn leaves: **(A)** Fall Armyworm. **(B)** Gray Leaf Spot. **(C)** Leaf Blight. **(D)** Leaf Healthy. **(E)** Northern Leaf Spot. **(F)** Rust Leaf.

The corn disease data for this paper comes from four different sources. We obtained images of three types of corn diseases images from the CD&S (Ahmad et al., 2021) dataset, namely northern leaf blight (NLB), gray leaf spot (GLS), and northern leaf spot (NLS) respectively. We obtained images of corn rust leaf (RL) under real conditions from the PlantDoc (Singh et al., 2020) dataset. Added to that, we got images of corn leaf infected by fall armyworm on a public website (https://github.com/FXD96/Corn-Diseases). Finally, we collected images of healthy corn leaves (LH) and a few images of other diseases through web crawler technology. **Figure 1** shows a sample corn disease dataset in a real environment.

### 2.2 Image preprocessing

In this paper, we collected 3258 images, including 432 images of Fall Armyworm, 613 images of Gray Leaf Spot, 688 images of Leaf Blight, 537 images of Leaf Healthy, 551 images of Northern Leaf Spot, 437 images of Rust Leaf. After that, the image data of each category are assigned to the training set, validation set and test set in the ratio of 8:1:1 respectively. **Table 1** shows the data distribution of corn leaf disease images.

**Table 1:** Distribution of image data by category.

Disease classes	Train	Valid	Test
Fall Armyworm (FA)	346	43	43
Gray Leaf Spot (GLS)	491	61	61
Leaf Blight (LB)	550	69	69
Leaf Healthy (LH)	429	54	54
Northern Leaf Spot (NLS)	441	55	55
Rust Leaf (RL)	351	43	43
Total	2608	325	325

**Figure 2:**
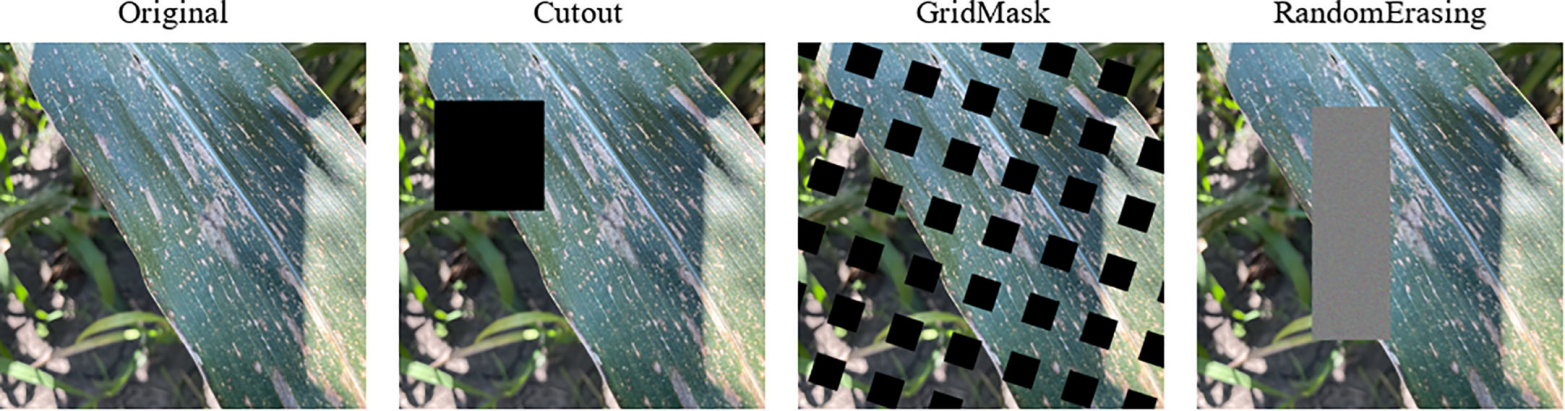
Classical online data augmentation. The Cutout method randomly cuts out part of the sample and fills it with 0 pixel values. Grid Mask generates a mask with the same resolution as the original image, and then multiplies that mask with the original image. Random Erasing randomly selects a region and then overwrites it with a random value.

In order to obtain better generalization and robustness and avoid overfitting problems, DL models usually need a lot of data as support (Lin et al., 2023). Therefore, three different online data augmentation methods are used in this paper, namely Cutout (DeVries and Taylor, 2017). Grid Mask (Chen et al., 2020) and Random Erasing (Zhong et al., 2020). **Figure 2** illustrates the online data enhancement method used in this paper.

## 3 Proposed method

**Figure 3:**
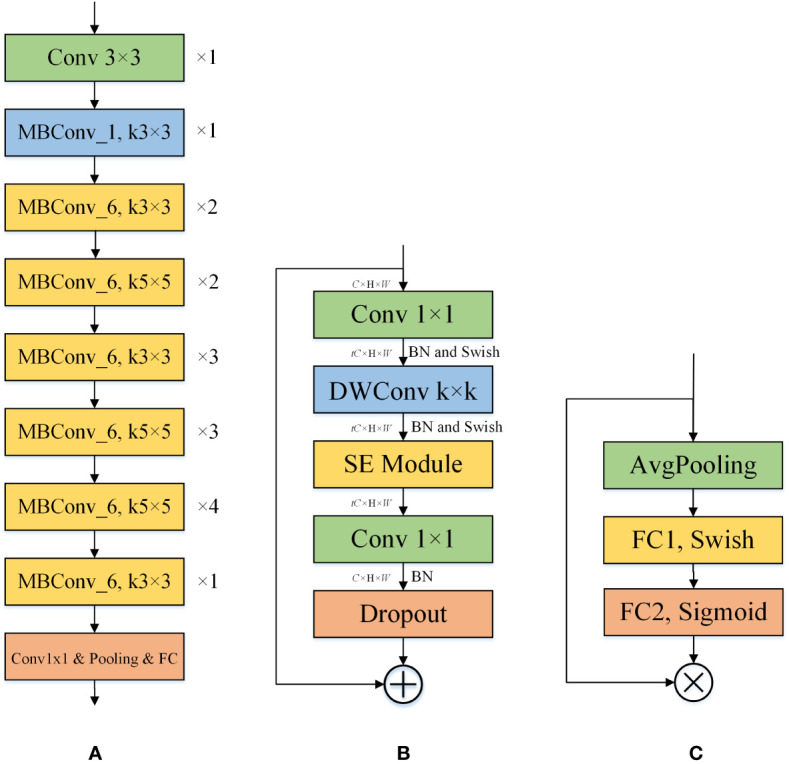
The individual structures included in EfficientNet-B0. **(A)** The structure of the EfficientNet-B0. ×n indicates that the layer is repeated n times, and kn×n indicates that the size of the convolution kernel is n. **(B)** The structure of the MBConv_t. k denotes the size of the convolution kernel of the DWConv layer. t indicates that the input channel is expanded by a factor of t. **(C)** The structure of the SE. SE can perform channel feature enhancement on the input feature map to improve the performance of the model by learning the relationship between different channels.

EfficientNet (Tan and Le, 2019) is a network series published by Google in May 2019, designed to improve model accuracy while keeping the model parameter count relatively low. Taking into account the deployment of the network on mobile devices, we chose the EfficientNet-B0 architecture and improved on it. It consists of nine stages, which comprise convolutional layers, MBConv1 layers, MBConv6 layers, pooling layers, and a fully connected layer. The overall architecture of EfficientNet-B0 is shown in **Figure 3**.

**Figure 4:**
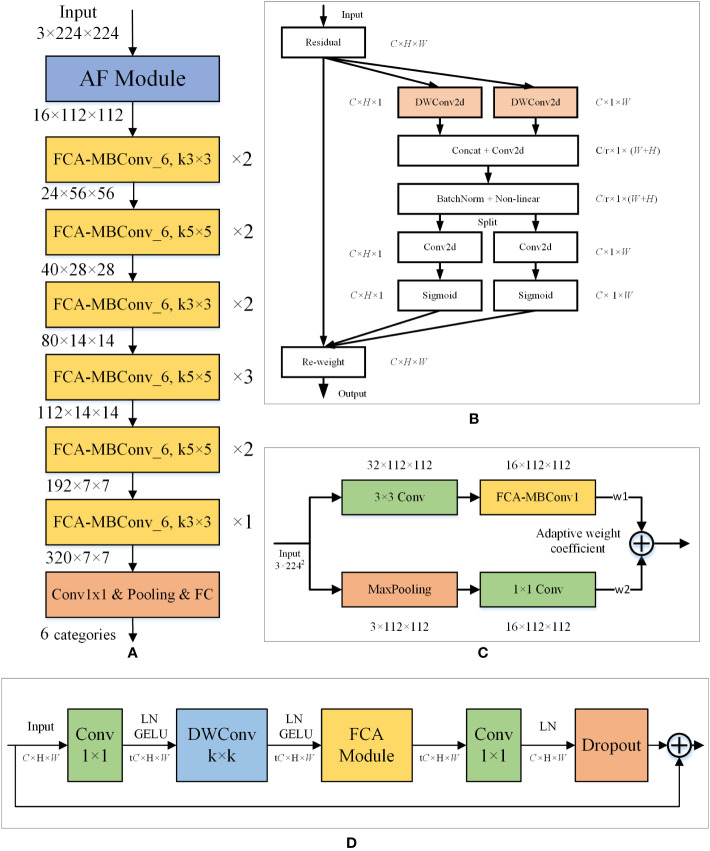
Layered architecture diagram of the FCA-EfficientNet. **(A)** The structure of the FCA-EfficientNet. ×n indicates that the layer is repeated n times, and kn×n indicates that the size of the convolution kernel is n. **(B)** The structure of the FCA module. Attentional information about spatial orientation is extracted through a convolutional model, allowing the network to better focus on the disease region and reduce interference from complex backgrounds. **(C)** The structure of the AF module. The AF module uses maximum pooling and 1×1 convolution, which reduces the interference of complex backgrounds and allows the model to better extract shallow features. **(D)** The structure of the FCA-MBConv_t. k denotes the size of the convolution kernel of the DWConv layer. t indicates that the input channel is expanded by a factor of t.

To identify corn disease images more accurately, we propose a new model based on the EfficientNet-B0 architecture, named FCA-EfficientNet. **Table 2** provides a detailed layer-by-layer description of FCA-EfficientNet. The overall architecture of FCA-EfficientNet is shown in **Figure 4**.

**Table 2:** Detailed configuration information for each layer of FCA-EfficientNet.

Stage *i*	Operator $\hat{F_{i}}$	Resolution $\hat{H_{i}}\times \hat{W_{i}}$	#Channels $\hat{C_{i}}$	#Layers $\hat{L_{i}}$	Dropout factor
1	Conv3×3	224×224	32	1	0
2	AF & FCA-MBConv1, k3×3	112×112	16	1	0.0125
3	FCA-MBConv6, k3×3	112×112	24	2	0.025
4	FCA-MBConv6, k5×5	56×56	40	2	0.05
5	FCA-MBConv6, k3×3	28×28	80	2	0.075
6	FCA-MBConv6, k5×5	14×14	112	3	0.1
7	FCA-MBConv6, k5×5	14×14	192	2	0.1375
8	FCA-MBConv6, k3×3	7×7	320	1	0.1625
9	Conv1×1 & Pooling & FC	7×7	1280	1	0

### 3.1 Fully-convolution-based coordinate attention

**Figure 5:**
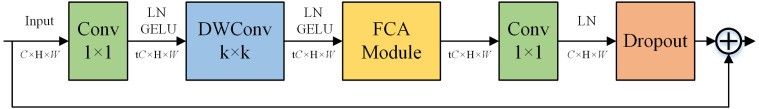
The structure of the FCA-MBConv. k denotes the size of the convolution kernel of the DWConv layer. t indicates that the input channel is expanded by a factor of t.

In order to avoid the interference of complex background, we replace the SE module in the MBConv structure with the FCA module, which enables the network to better focus on the lesion area. **Figure 5** shows the overall structure of the FCA-MBConv module.

**Figure 6:**
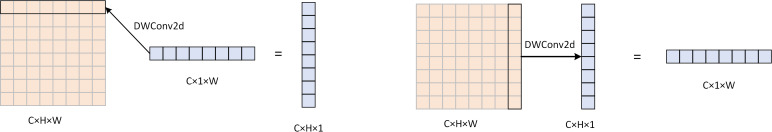
The computational procedure of DWConv2d, using a DW convolution of 1 × W, can obtain attention in the width direction of size H × 1 by focusing on the feature information in the width direction of the picture. Similarly, using DW convolution of H × 1, attention in the height direction of size 1 × W can be obtained.

**Figure 7:**
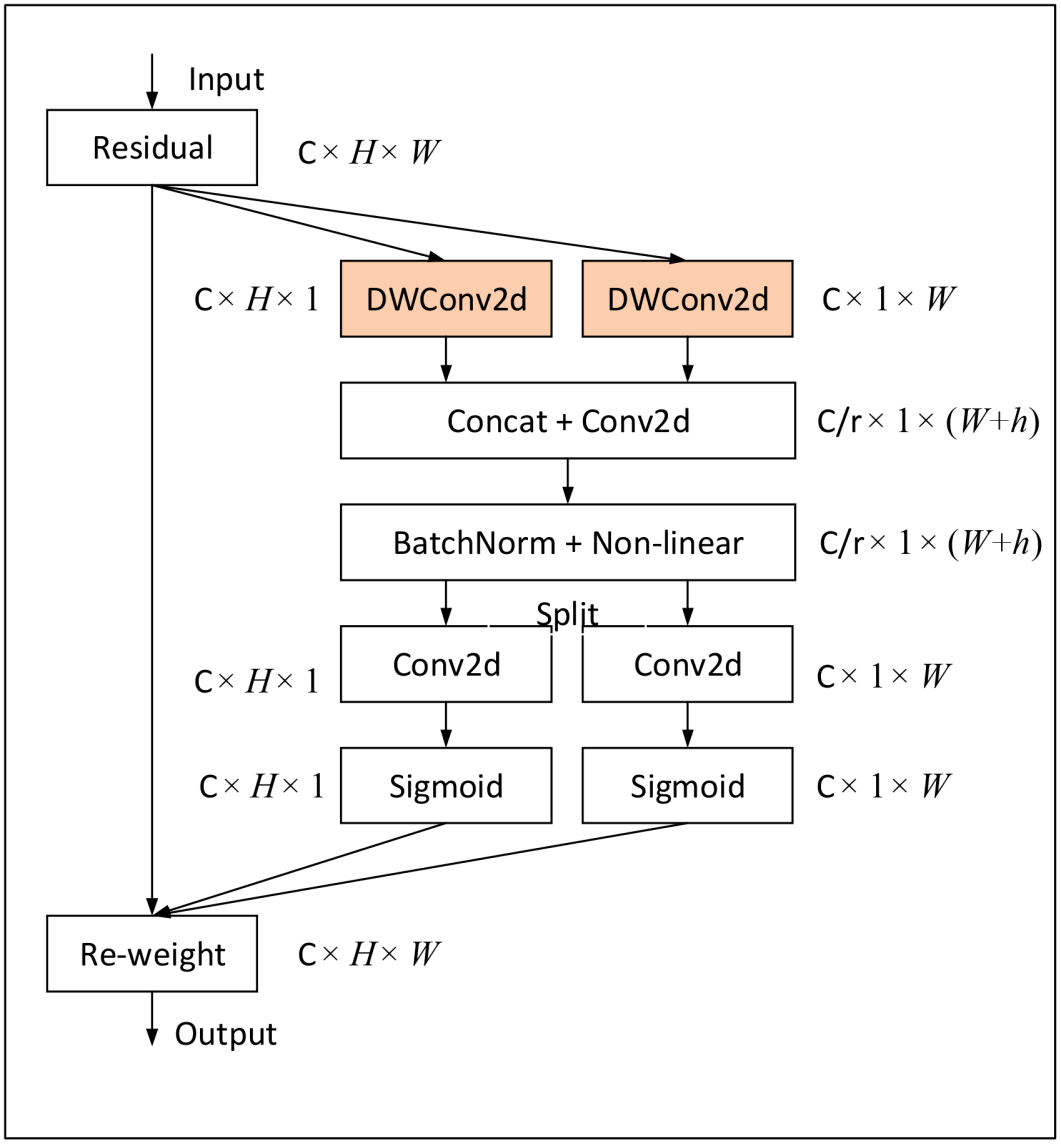
The structure of the FCA module. We use DWConv 2d to replace the average pooling layer in CA, which is able to capture critical disease information through convolutional operations while being less susceptible to interference from complex backgrounds.

FCA module based on Coordinate Attention (CA) (Hou et al., 2021). While CA employs global average pooling to aggregate horizontal and spatial features, this approach fails to capture critical disease information in the feature map for fine-grained recognition tasks, such as corn disease recognition, leading to recognition accuracy bottlenecks. To overcome this limitation, we remove the global average pooling layer and employ a convolutional kernel with a size of horizontally, and a convolutional kernel with a size of vertically, as shown in **Figure 6**. This modification enables the attention module to automatically capture salient disease information in different spatial directions and aggregate it, thereby strengthening the ability to capture dependencies between remote disease feature information while preserving precise location information of disease features. The structure of the FCA module is shown in **Figure 7**.

Assuming the input feature map is *x_c_*, where *c* denotes the number of feature map channel. a new feature map ${\hat{x}}_{c}$ can be obtained by two convolution operations for feature extraction.


(1) $${\hat{x}}_{tc}=DWConv_{tc}^{k\times k}(Conv_{tc}^{1\times 1}(x_{c}))$$

Convolution of $\hat{x}$ in the horizontal and vertical directions separately. This results in two output feature maps.


(2) $$Z_{tc}^{h}(h)=Conv^{1\times W}({\hat{x}}_{tc})$$

(3) $$Z_{tc}^{w}(w)=Conv^{H\times 1}({\hat{x}}_{tc})$$

The two output feature maps are concatenated along the channel dimension and passed through a 1x1 convolutional layer $Conv^{1\times 1}$ followed by a non-linear activation function *δ* to obtain the output feature map *f*.


(4) $$f=\delta (Conv^{1\times 1}(concat\left\{z^{h};\right.\left.z^{w}\right\}))$$

*f* is decomposed into two outputs along the spatial dimension and transformed into tensors with the same number of channels as the input by two 1×1 convolutions (g and h). The two outputs can be represented by *g^h^* and *g^w^*:


(5) $$g^{h}=Sigmoid(Conv_{h}^{1\times 1}(f^{h}))$$

(6) $$g^{w}=Sigmoid(Conv_{w}^{1\times 1}(f^{w}))$$

the output expression of the FCA module is:


(7) $$y_{FCA}(i,j)={\hat{x}}_{tc}(i,j)\times g_{tc}^{h}(i)\times g_{tc}^{w}(j)$$

Finally, the feature dimensions are reduced and residual joins are performed through the Conv layer, and the output of the FCA-MBConv module is:


(8) $$MBConv_{F}{{\;}_{C}}_{A}(x_{c})=x_{c}+Dropout(Conv_{c}^{1\times 1}y_{FCA}({\hat{x}}_{tc}))$$

### 3.2 Adaptive fusion

**Figure 8:**
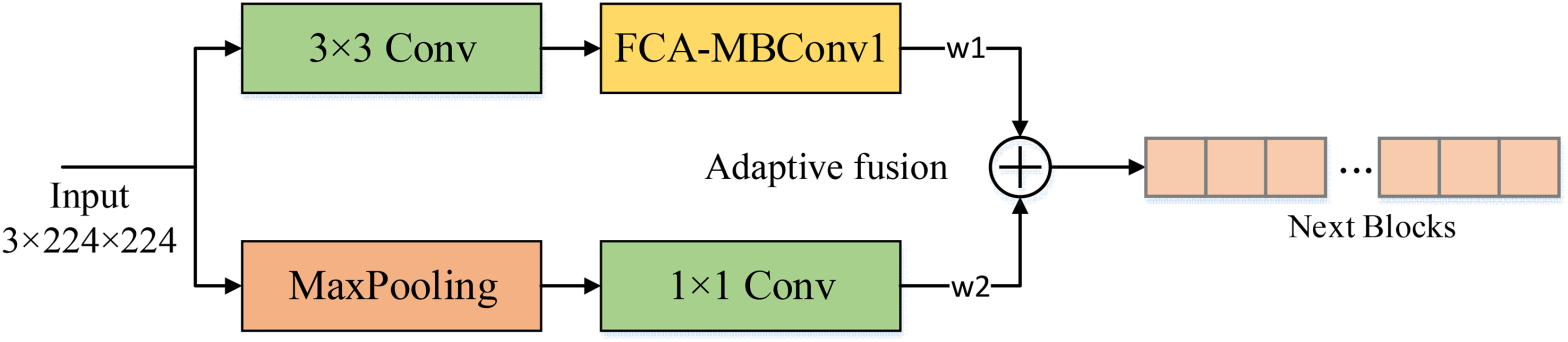
The structure of the AF module. The noise is filtered out using the maximum pooling layer and then dynamically introduced into the backbone network by adaptive parameters to reduce the effect of background noise on the model. w1, w2 are learnable weight coefficients.

In real-world crop disease images, the presence of complex background noise often makes it challenging to extract shallow features using convolutional layers alone, which can significantly affect the recognition accuracy of the model. To address this issue, we introduced a max-pooling layer to assist in shallow feature extraction. Unlike the conventional feature fusion approaches that involve simple addition or concatenation, we adopted two learnable weighting coefficients to evaluate the feature maps derived from the two methods. This approach enables the model to dynamically allocate weights to the pooled and convolved feature maps based on the complexity of the background information, resulting in effective extraction of shallow convolutional layer features while avoiding interference from complex background noise. As a result, the effectiveness and robustness of the model is significantly improved. The structure of the AF module is shown in **Figure 8**.

Assuming the input image is *x*, Two output *y_m_*,*y_f_* can be obtained by max pooling and backbone. We use the learnable weight parameters *w*_1_,*w*_2_ to weight the two and obtain the final output feature map *y_AF_*:


(9) $$y_{m}=Conv^{1\times 1}(Maxpool^{2\times 2}(x))$$

(10) $$y_{f}=MBConv_{FCA}(Conv_{3\times 3}(x))$$

(11) $$y_{A}{\;}_{F}=w_{1}\times y_{m}+w_{2}\times y_{f}$$

### 3.3 Layer normalization and gaussian error linear unit

In CNN models, normalization techniques are used to adjust the distribution of the input data to better fit the training process of the neural network. The formula for normalization is as follows.


(12) $$f(x)=\frac{x-E[x]}{\sqrt{Var[x]+ò}}*\gamma +\beta$$

where *x* denotes the input feature map and *f*(*x*) the output feature map after normalization.

**Figure 9:**
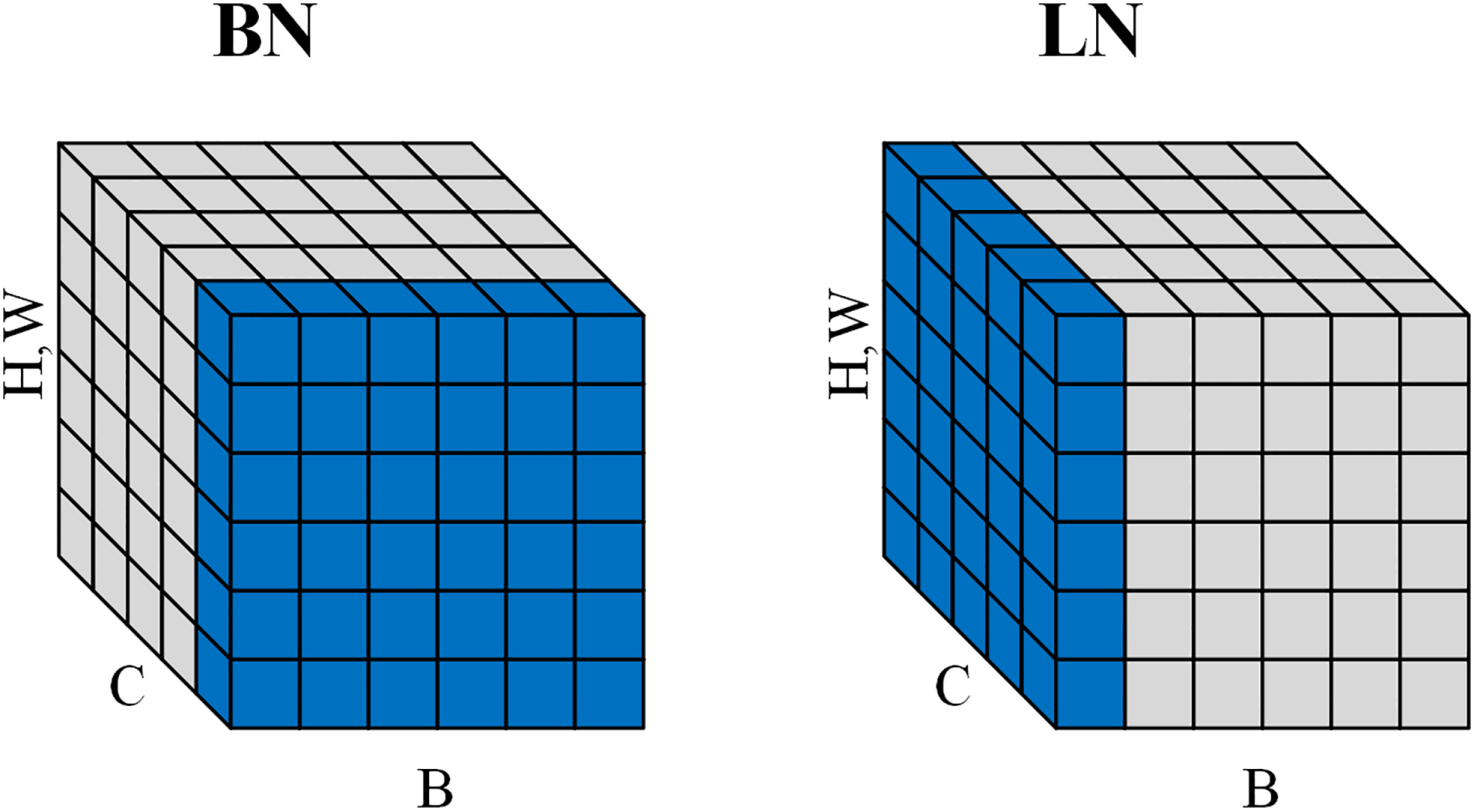
Normalization directions for BN and LN. BN does normalization along the batch direction and calculates the mean of B×H×W. LN does normalization along the channel direction and calculates the mean of C×H×W.

The use of batch normalization (BN) (Ioffe and Szegedy, 2015) in EfficientNet allows the model to accelerate convergence during training, but the inconsistency of BN between training and testing may adversely affect the model performance. Therefore, we adopt layer normalization (LN) (Ba et al., 2016) instead of BN, which has the same performance during training and testing and is easier to implement. LN normalizes the output of each convolutional layer to a normalized value equal to the mean and variance of the inputs, which reduces the memory consumption during the training of the model and improves the generalization ability of the model. As shown in **Figure 9**. Unlike BNs that are normalized in the batch direction, LN pairs are normalized in the channel direction. This makes LN independent of batch size.

Also to speed up the convergence of the model, we used gaussian error linear unit (GELU) (Hendrycks and Gimpel, 2016) to replace Swish (Ramachandran et al., 2017). Their formulas are as follows.


(13) $$Swish(x)=x\cdot sigmoid(x)=\frac{x}{1-e^{-}{\;}^{x}}$$

(14) GELU(x)=x×P(X<=x)=x×ϕ(x),x~N(0,1)

**Figure 10:**
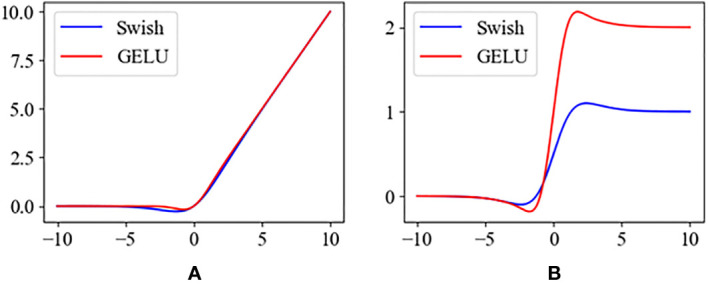
**(A)** The activation functions of the Swish and the GELU. **(B)** The derivatives of Swish and GELU.

The plots of Swish and GELU functions and their derivatives are shown in **Figure 10**. Compared to Swish, GELU tends to converge faster, which makes it more suitable for model training.

### 3.4 Optimal architecture

Multiple MBConv layers are included in EfficientNet, and by simply repeating the MBConv layers, the model can be made to learn complex features and achieve better performance. However, EfficientNet was trained by a large dataset like ImageNet with a large number of samples and categories, compared to the corn disease dataset we used with fewer samples and categories. As a result, there are some redundant structures in the original network, and although the effect of these redundant structures on the accuracy can be reduced by the residual structures, these structures cause the model’s to perform unnecessary computations, which affects the recognition speed of the model. Therefore, we remove these redundant structures to reduce the number of parameters of the model and increase the speed of computation without affecting the accuracy of model recognition, which helps in deployment on mobile devices.

## 4 Experimental results and analysis

### 4.1 Evaluation indexes

Accuracy (Acc), Precision (P), Recall (R), F1 score (F1) are utilized as the evaluation metrics in this study, they are defined as follows:


(15) $$Acc=\frac{TP+TN}{TP+TN+FP+FN}$$

(16) $$P=\frac{TP}{TP+FP}$$

(17) $$R=\frac{TP}{TP+FN}$$

(18) $$F1=\frac{P\times R}{P+R}\times 2$$

Where TP, TN, FP, and FN are the abbreviations used to represent the count of true positive, true negative, false positive, and false negative samples, respectively. Furthermore, the parameter count of the model is also considered to evaluate its performance.

### 4.2 Experimental configuration and hyperparameter setting

This study utilized the language of python-3.7.13 and pytorch-1.12.1 deep learning frameworks for experimentation. All experiments were conducted on a Windows 10 operating system, with an Intel(R) Xeon(R) W-1390 CPU and an NVIDIA RTX3090 GPU. The hyperparameters for model training were set to the configurations listed in **Table 3** for all models.

**Table 3:** Hyperparameter configuration for model training.

Parameter	Value
Optimizer	Adam
Loss Function	Cross-Entropy
Training Epochs	200
Warmup Epochs	20
Learning Rate	0.001
ExponentialLR Gamma	0.99
Batch Size	32

### 4.3 Performance of FCA-EfficientNet

**Figure 11:**
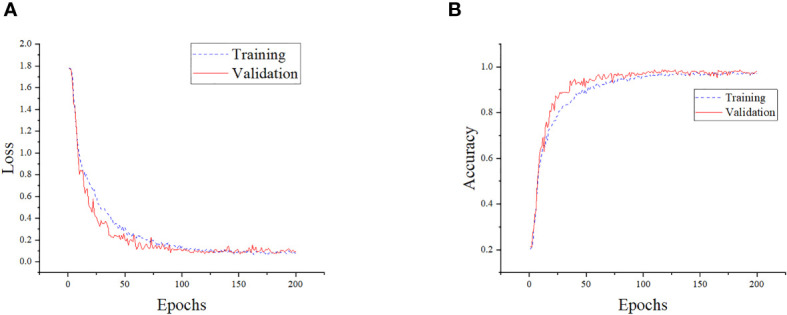
Training details of the model. **(A)** Training loss and validation loss of the model. **(B)** Training accuracy and validation accuracy of the model.

The performance of the model on the training and validation sets is shown in **Figure 11**. To assess the recognition performance of the model, we conducted a separate analysis of its recognition capabilities on various categories of disease images in the test set. By employing a comprehensive set of evaluation metrics, we performed a quantitative analysis and the experimental results are presented in **Table 4**. As shown in the table, our model achieved an accuracy of up to 100% in identifying various types of corn diseases, with none falling below 95%. This demonstrates the model’s precise recognition of corn diseases. Notably, the model’s performance in identifying Northern Leaf Spot images was exceptional, with all evaluation metrics reaching the highest levels. Conversely, the model’s recognition performance on Rust Leaf images was comparatively lower, possibly due to the similarity to images of other categories. Additionally, the average precision, recall, and F1 scores of 98.63%, 98.76%, and 98.68%, respectively, demonstrate the model’s superior overall performance, making it highly effective in accomplishing the corn disease classification task.

**Table 4:** Evaluation results of FCA-EfficientNet on corn images of different categories.

Categories	Precision	Recall	F1	TP	FP	TN	FN
Fall Armyworm	0.9773	1.0	0.9885	43.0	1.0	0.0	281.0
Gray Leaf Spot	1.0	0.9672	0.9833	59.0	0.0	2.0	264.0
Leaf Blight	0.9857	1.0	0.9928	69.0	1.0	0.0	255.0
Leaf Healthy	1.0	0.9815	0.9907	53.0	0.0	1.0	271.0
Northern Leaf Spot	1.0	1.0	1.0	55.0	0.0	0.0	270.0
Rust Leaf	0.9545	0.9767	0.9655	42.0	2.0	1.0	280.0

**Figure 12:**
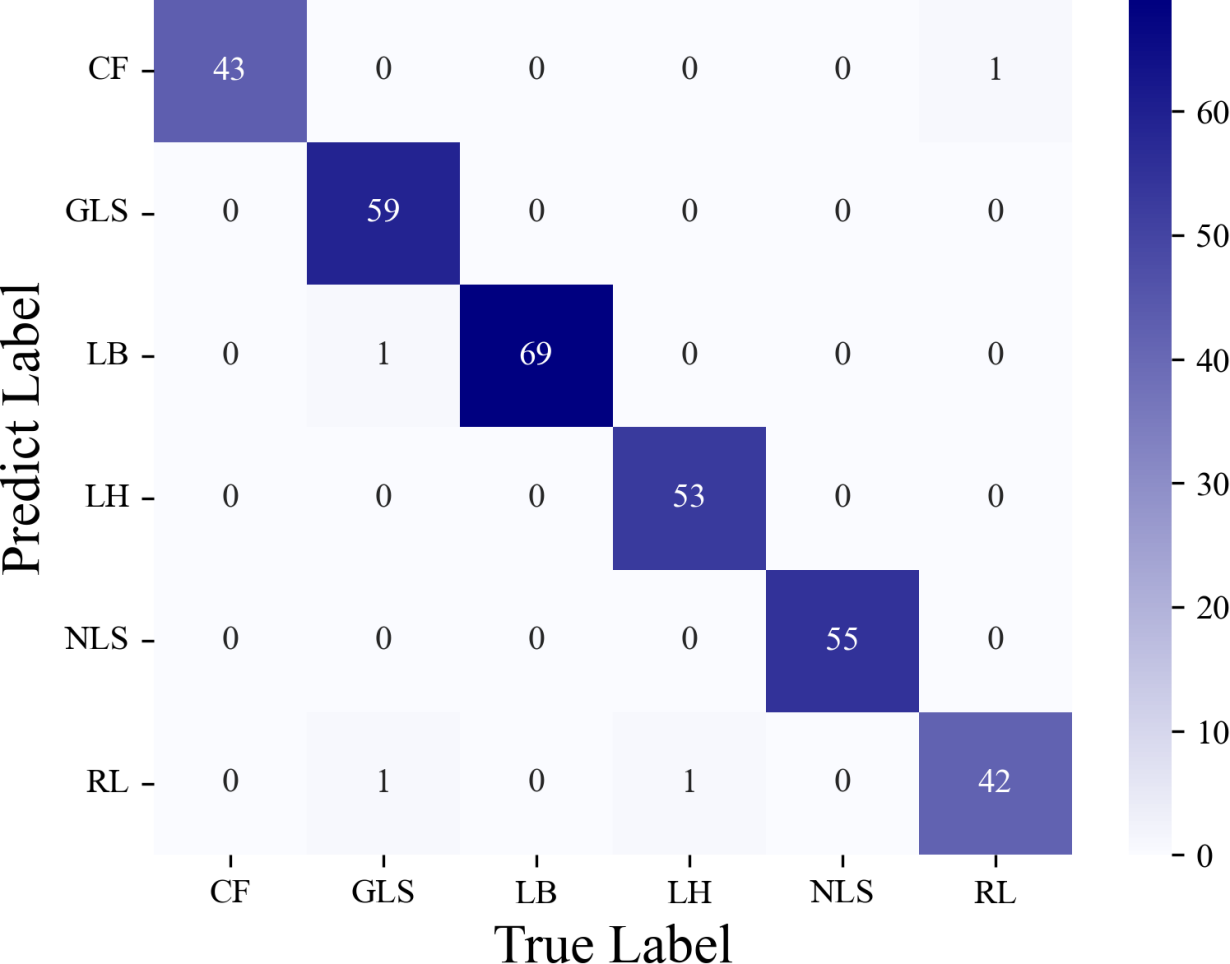
The confusion matrix of classification results for corn diseases on the test set.

In **Figure 12**, a confusion matrix is presented to visualize the recognition performance of the designed model on corn disease images. The matrix displays the true and predicted classes of the data along its horizontal and vertical axes, respectively, with the diagonal entries indicating the number of correct predictions. The confusion matrix suggests that the proposed model is capable of effectively identifying multiple classes of corn disease images, as the majority of images in the provided dataset are classified correctly. The misclassifications observed may be attributed to small inter-class differences between certain categories and background interference. Notably, it achieves a high degree of accuracy in correctly classifying all disease images of the GLF, LH, and NLS categories, while maintaining a low rate of false positives for images of other categories. These results underscore the model’s high precision and reliability for identifying corn diseases.

### 4.4 Comparison of different nodes for adaptive fusion

**Figure 13:**
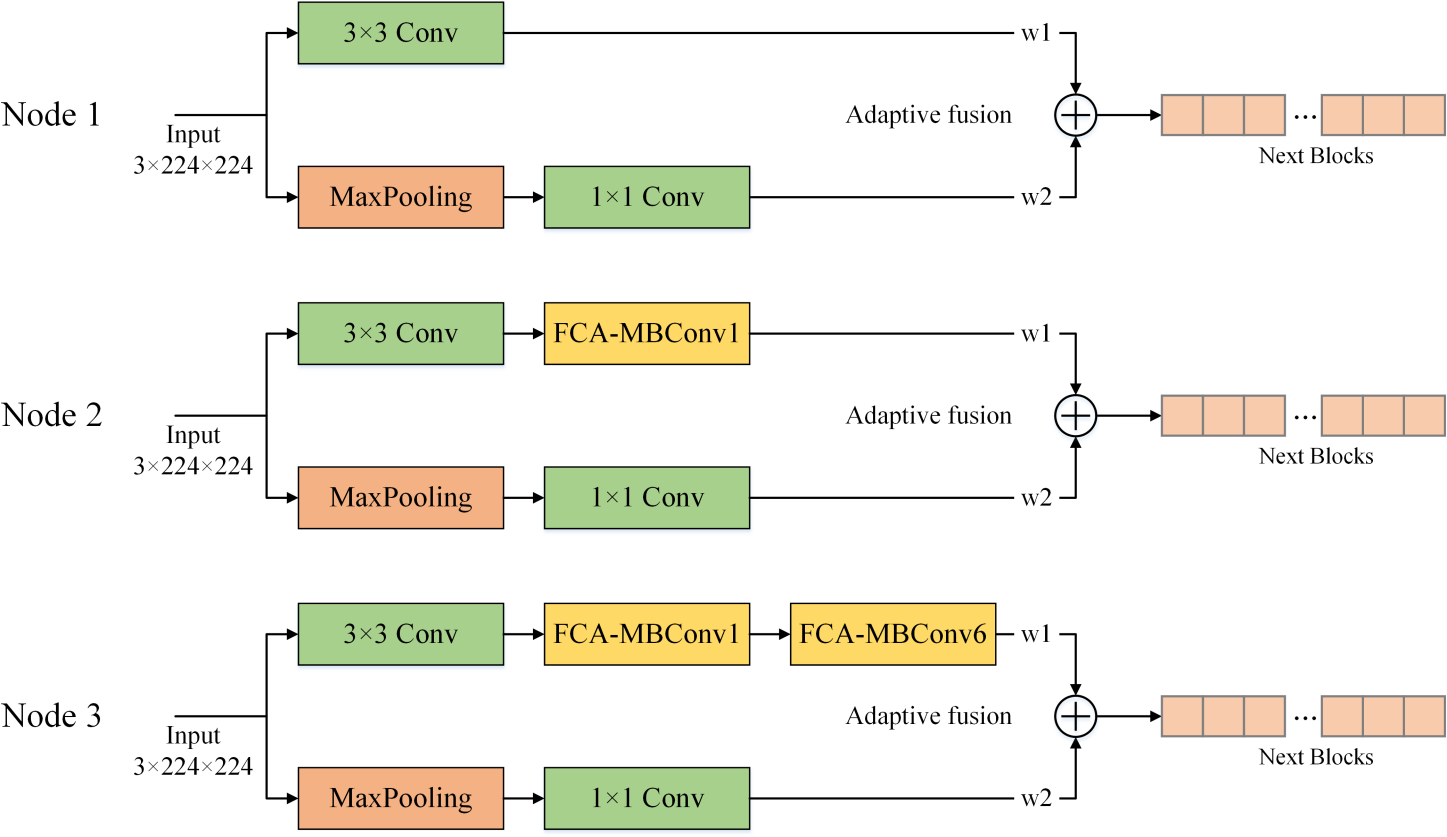
Different nodes for adaptive feature fusion. Node 1 is fused after the convolution layer, node 2 is fused after the first FCA-MBConv extracted features, and node 3 is fused after the second FCA-MBConv layer.

In order to determine the optimal fusion location, we conducted experiments on three different nodes in the shallow network: after the first convolutional layer, after the first FCA-MBConv module, and after the second FCA-MBConv module. **Figure 13** shows different fusion nodes. The fusion nodes were selected based on their potential to improve the model’s performance. The results, as presented in **Table 5**, indicate that the addition of AF at Node1, Node2, and Node3 led to improvements in various evaluation metrics, while maintaining a similar parameter count. Notably, applying AF to the shallow layers of the network resulted in better recognition results compared to the deep layers. Specifically, the performance improvement was most significant when applying AF to Node2, resulting in a 0.92% increase in recognition accuracy and a 0.91% increase in F1-score. These findings suggest that incorporating AF into specific locations of the network can effectively enhance its performance.

**Table 5:** The AF for the different nodes.

Model	Accuracy	Precision	Recall	F1	Param
EfficientNet (FCA)	0.9631	0.9650	0.9603	0.9619	4.02 M
AF on Node1	0.9692	0.9709	0.9670	0.9682	4.02 M
AF on Node2	0.9723	0.9726	0.9698	0.9710	4.02 M
AF on Node3	0.9631	0.9611	0.9593	0.9592	4.02 M

### 4.5 Comparison of different attention mechanisms

Different attention mechanisms have a significant impact on the performance of CNN models, and adding suitable attention mechanisms can optimize recognition results. This study compared and analyzed the effects of three attention mechanism modules, namely SE, CA, and our proposed FCA on model performance. As shown in the **Table 6**, adding the CA module did not significantly improve the overall recognition performance of the model, and the F1 score even decreased. However, after adding the FCA module, the model’s performance was significantly improved, with an increase in recognition accuracy of 0.93%. This result verifies that adding the FCA module can effectively promote the model’s focus on disease information, leading to better recognition results.

**Table 6:** The effects of different attention mechanisms.

Attention Mechanism	Accuracy	Precision	Recall	F1	Param
AF-EfficientNetB0 (SE)	0.9723	0.9726	0.9698	0.9710	4.02 M
AF-EfficientNetB0 (CA)	0.9723	0.9728	0.9701	0.9711	4.79 M
AF-EfficientNetB0 (FCA)	0.9816	0.9848	0.9837	0.9841	5.00 M

**Figure 14:**
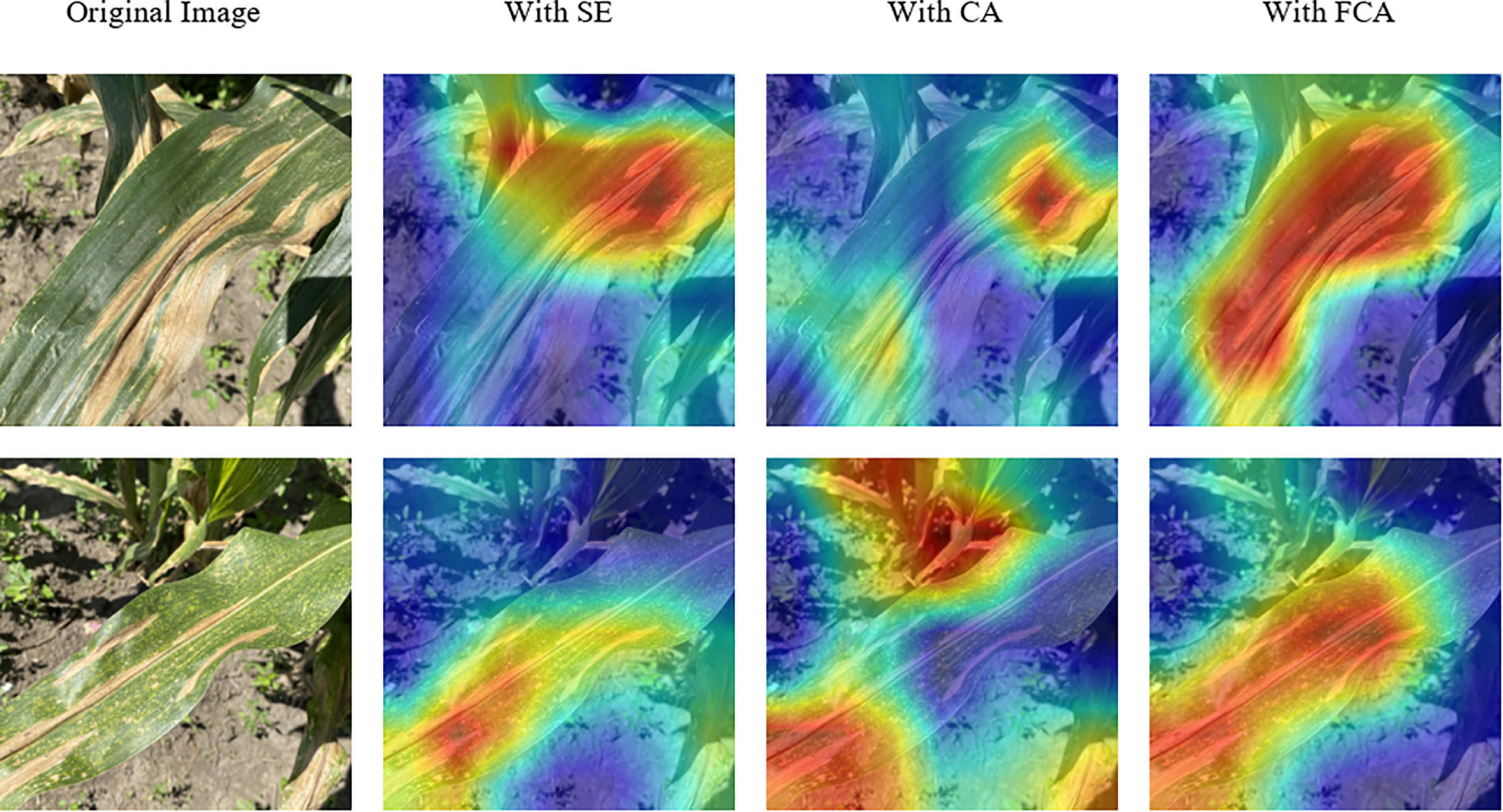
Visualization results of models with different attention mechanisms. The red regions indicate the areas where the model focuses on.

To analyze the learning ability of models with different attention mechanisms on disease information, we used Grad-Cam (Selvaraju et al., 2017) to generate class activation maps for visualization, as shown in **Figure 14**. The first column shows the original disease image, and the other columns show the visualization results of different models. The results in the first row of the figure demonstrate that the use of the FCA module enables the model to more comprehensively focus on the primary diseased regions. Moreover, the comparative results presented in the second row of the figure reveal significant disparities between the models. Specifically, the model utilizing the SE module inadequately attends to the entire diseased area, while the model leveraging the CA module primarily focuses on background information. Conversely, the FCA module precisely localizes the distinctive diseased feature region, underscoring its ability to promote the model’s attention to disease information. The results indicate that the FCA module can promote the model’s attention to disease information and is more suitable for identifying corn diseases.

### 4.6 Comparison of different normalization methods and activation function

In order to investigate the impact of different normalization methods and activation functions on the model, we conducted comparative analysis experiments by combining BN, LN, SiLU, and GELU. As shown in **Table 7**, replacing the BN with the LN reduces the complexity of the model, as evidenced by the reduction in Flops, while the parameter counts remain relatively unchanged. In addition, the combination of LN and GELU yields the highest recognition accuracy and precision, and sub-optimal recall and F1-score. The consistency of LN’s performance in training and testing, and the approximately 3% reduction in Flops, make it more suitable for model deployment.

**Table 7:** The effects of different combinations.

Method	Accuracy	Precision	Recall	F1	Param	Flops
BN & Swish	0.9816	0.9848	0.9837	0.9841	5.00 M	420.23M
BN & GELU	0.9815	0.9822	0.9802	0.9806	5.00M	420.23M
LN & Swish	0.9785	0.9782	0.9771	0.9775	5.05M	407.68M
LN & GELU	0.9846	0.9857	0.9818	0.9831	5.05M	407.68M

### 4.7 Optimal architecture

We removing some redundant layers in Stages 5, 6, and 7, the performance of all networks on the test set is shown in the following **Table 8**. From the table, it can be observed that removing only one redundant layer does not significantly decrease the performance of the model (rows 6, 9, and 11 in the table). The model can still maintain good performance even after removing all redundant layers (row 1 in the table). However, removing all redundant layers in shallow stages while keeping the same number of layers in Stage 7 may lead to overfitting and a significant decrease in model performance (row 3 in the table). With consideration of the accuracy, parameter quantity, and computational complexity of the models, we finally selected 2 layers in Stage 5, 3 layers in Stage 6, and 2 layers in Stage 7 (row 4 in the table).

**Table 8:** The performance of different layers on the test set.

Row	Layers (Stage 5, 6, 7)	Accuracy	Precision	Recall	F1	Param	Flops
1	2, 2, 2	0.9815	0.9826	0.9796	0.9806	3.17M	305.1M
2	2, 2, 3	0.9846	0.9854	0.9842	0.9846	3.90M	330.62M
3	2, 2, 4	0.9754	0.9763	0.9738	0.9748	4.64M	356.15M
**4**	**2, 3, 2**	**0.9877**	**0.9863**	**0.9876**	**0.9868**	**3.44M**	**339.74M**
5	2, 3, 3	0.9846	0.9840	0.9853	0.9844	4.18M	365.26M
6	2, 3, 4	0.9815	0.9808	0.9818	0.9811	4.91M	390.79M
7	3, 2, 2	0.9846	0.9846	0.9845	0.9845	3.31M	321.99M
8	3, 2, 3	0.9785	0.9808	0.9776	0.9790	4.04M	347.52M
9	3, 2, 4	0.9846	0.9870	0.9849	0.9857	4.78M	373.04M
10	3, 3, 2	0.9846	0.9833	0.9844	0.9838	3.58M	356.64M
11	3, 3, 3	0.9815	0.9805	0.9806	0.9801	4.31M	382.16M
12	Origin (3,3,4)	0.9846	0.9857	0.9818	0.9831	5.05M	407.68M

Bold indicates the optimal structure used in this paper.

### 4.8 Ablation experiment

This study conducted ablation experiments to demonstrate the efficacy of the proposed model in improving model performance. **Table 9** presents the results of the ablation experiments on the test set. The introduction of the AF module, FCA module, LN and GELU led to accuracy improvements of 0.9%, 0.9%, and 0.3%, respectively. Moreover, we have identified the optimal neural network architecture that achieves a 0.3% improvement in accuracy on the test set while reducing the model’s parameter count. The improved model reduced the parameters by 0.58M compared to the original EfficientNet model and achieved a 2.4% increase in accuracy. These findings highlight the ability of the designed model to effectively learn disease feature information in corn disease images, thereby achieving superior recognition performance with lower parameter consumption following model optimization.

**Table 9:** Ablation experiments on the test set.

Method	Accuracy	Precision	Recall	F1	Param	Flops
EfficientNetB0	0.9631	0.9650	0.9603	0.9619	4.02M	398.03M
+AF	0.9723	0.9726	0.9698	0.9710	4.02M	399.38M
+ FCA	0.9816	0.9848	0.9837	0.9841	5.00M	420.23M
+LN & GELU	0.9846	0.9857	0.9818	0.9831	5.05M	407.68M
Architecture Search	0.9877	0.9863	0.9876	0.9868	3.44M	339.74M

### 4.9 Comparison with different models

**Figure 15:**
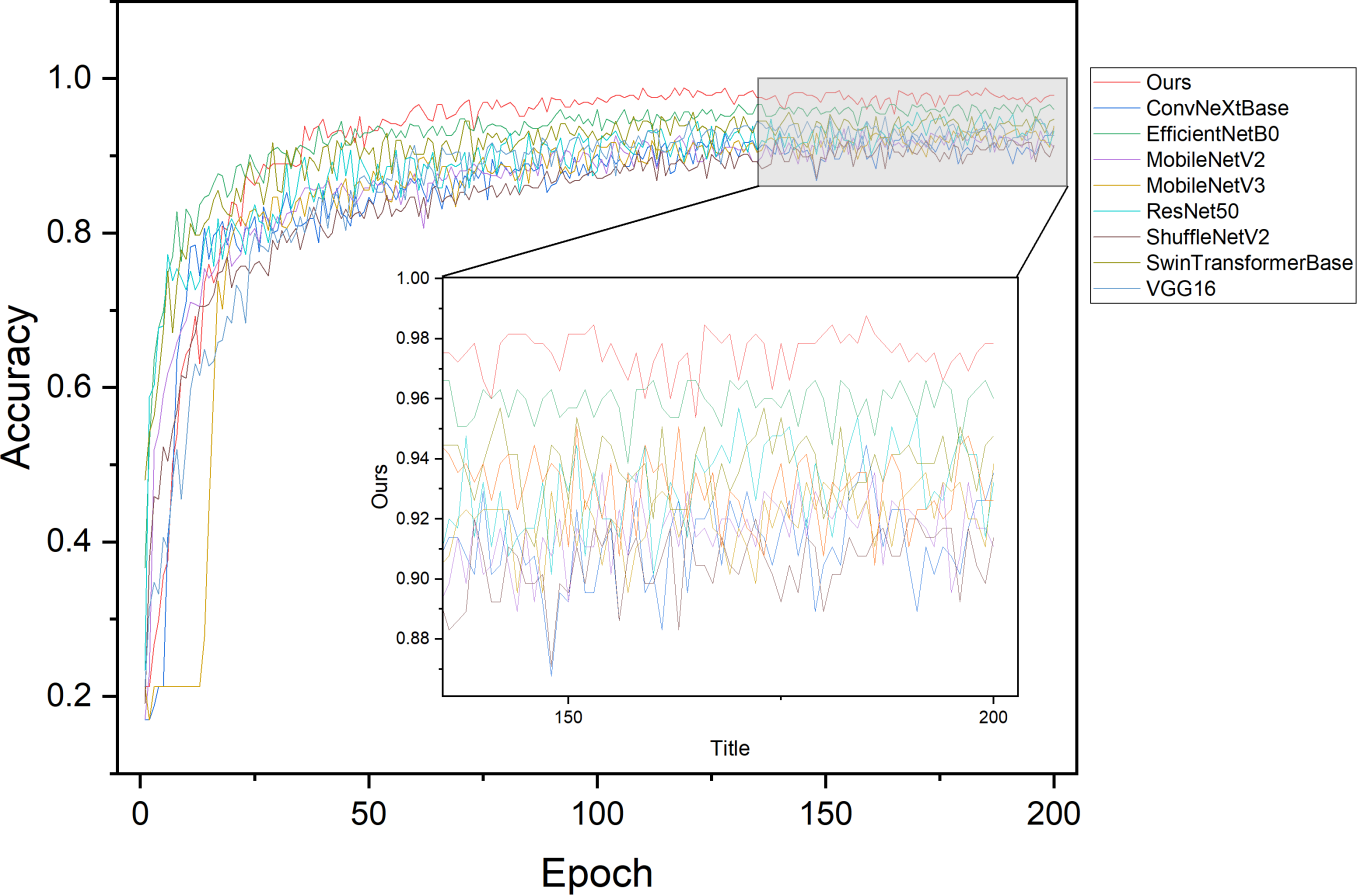
Validation accuracy curves of different models during training.

CNN has shown superior performance in the field of image classification, and CNN-based classification models have diversity for different domains. To demonstrate the classification performance of the designed model, this study conducted experiments on corn disease recognition using existing classic CNN models under the same training conditions. The experiments used different models with varying parameter sizes, including VGG16, ResNet50, Shufflenetv2, and MobileNetv2, and the accuracy change curves of each model on the validation set during 200 epochs of training are shown in **Figure 15**. It is not difficult to observe from the validation curve graph that, compared with existing classic models, the proposed model can achieve a higher accuracy at a faster rate and with less oscillation, demonstrating better accuracy and stability.

**Table 10** shows the recognition performance of different models on the test set, and compares and analyzes the advantages of the proposed model using metrics such as accuracy, precision, recall, F1-score, parameter count, Flops and inference time. Among the existing classic CNN models, VGG16, ResNet50, and DenseNet169 all have an accuracy of around 95%, while lightweight models such as MobileNet and ShuffleNet have lower accuracy at around 94%. In contrast, our model achieves an accuracy of 98.77% with only 3.44M parameters. Moreover, our model has precision, recall, and F1-score all greater than 98% with the lowest number of parameters, indicating strong generalization ability and significant advantages over other models, making it more suitable for the recognition task of corn disease images.

**Table 10:** Performance and parameter comparison of different models on the test set.

Model	Accuracy	Precision	Recall	F1	Param	Flops	Inference Time^1^
ConvNeXt-Base	0.9446	0.9467	0.9433	0.9446	87.52M	15.35G	12.096ms
DenseNet	0.9508	0.9539	0.9456	0.9475	6.96M	2.90G	14.316ms
EfficientNet-B0	0.9631	0.9650	0.9603	0.9619	4.02M	398.03M	9.490ms
MobileNetV2	0.9477	0.9545	0.9441	0.9467	2.23M	318.96M	6.847ms
MobileNetV3	0.9262	0.9235	0.9227	0.9213	1.52M	58.79M	4.672ms
ResNet50	0.9538	0.9581	0.9501	0.9531	23.52M	4.12G	6.422ms
ShuffleNetV2	0.9200	0.9191	0.9185	0.9178	1.26M	149.58M	5.675ms
SwinTransformer-Base	0.9569	0.9553	0.9550	0.9545	86.69M	15.17G	16.2103ms
VGG16	0.9508	0.9498	0.9487	0.9489	134.29M	15.5G	11.9590ms
**Ours**	**0.9877**	**0.9863**	**0.9876**	**0.9868**	**3.44M**	**339.74M**	**7.218ms**

Bold indicates the final performance of the network.

^1^Model's inference time tested on NVIDIA RTX3090

**Figure 16:**
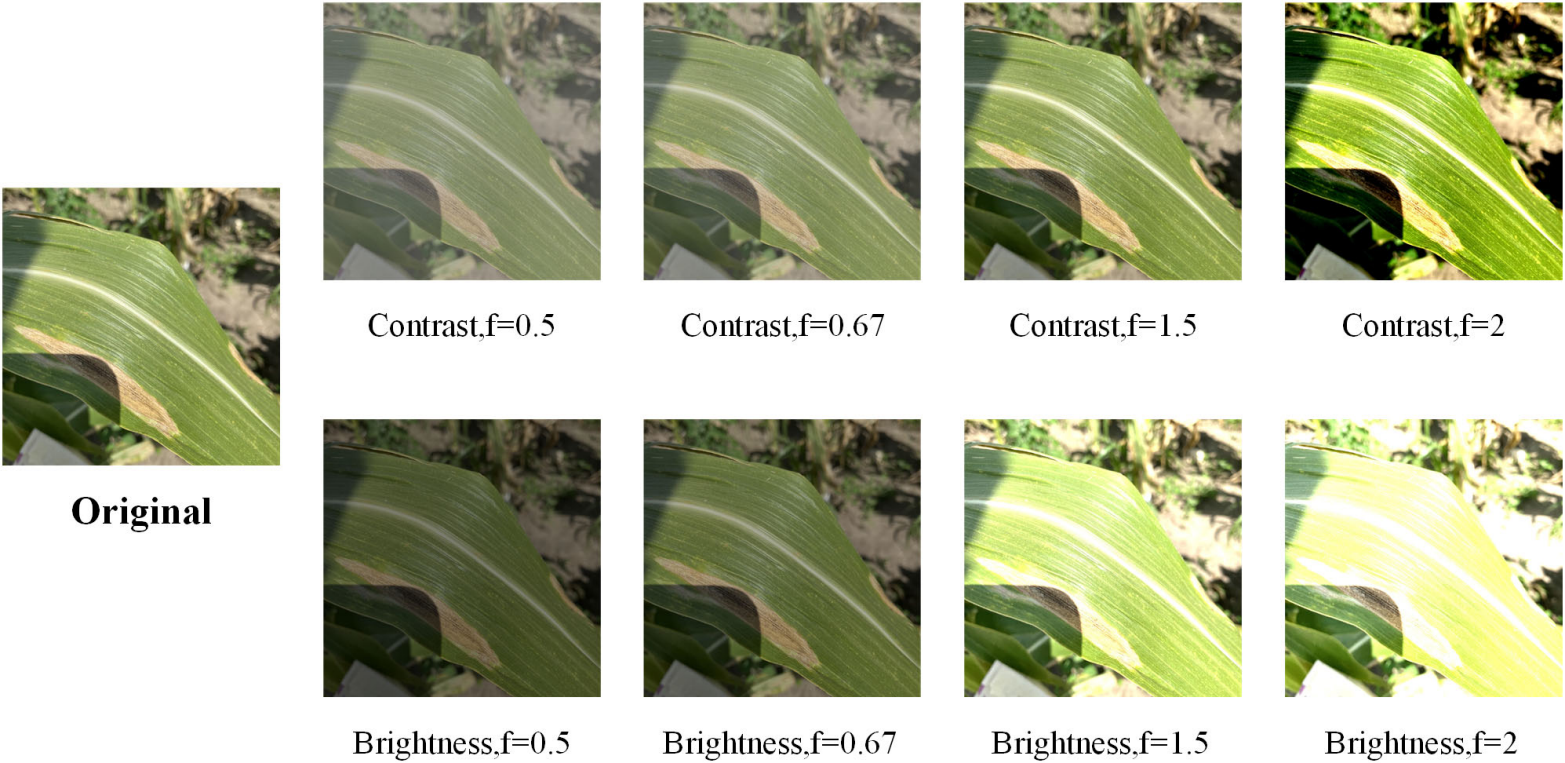
Sample image after enhancement of the test set. Where f denotes the enhancement factor.

Meanwhile, in order to further verified the robustness of the model, we changed the contrast and brightness of the test set, images to test the recognition accuracy of the model under different lighting conditions. We used four different magnifications (0.5, 0.67, 1.5, 2) to change the contrast or brightness of the image, respectively. The images before and after enhancement are shown in **Figure 16**.

**Table 11** lists the recognition accuracies of the different networks on the augmented test set, and it can be seen that the recognition accuracies of the networks all produce a large drop due to the substantial changes made to the images, but the recognition accuracies of our proposed network drop to a lesser extent compared to the other networks, which suggests that the model’s is more robust under different lighting conditions.

**Table 11:** Comparison of the performance of different models under different light conditions.

Model	Accuracy	Precision	Recall	F1
ConvNeXt-Base	0.6614	0.7005	0.6769	0.6724
DenseNet	0.6275	0.6958	0.6484	0.6345
EfficientNet-B0	0.6704	0.6866	0.6709	0.6662
MobileNetV2	0.5673	0.5888	0.5945	0.5739
MobileNetV3	0.6451	0.6823	0.6560	0.6498
ResNet50	0.9538	0.9581	0.9501	0.9531
ShuffleNetV2	0.5215	0.6043	0.5320	0.5296
SwinTransformer-Base	0.6924	0.7229	0.6936	0.6868
VGG16	0.6519	0.6847	0.6580	0.6444
**Ours**	**0.7389**	**0.7397**	**0.7489**	**0.7393**

Bold indicates the final performance of the network.

### 4.10 Comparison with relevant studies

To further evaluated the performance of the model, we compared it with some related studies that have been conducted. **Table 12** lists some of the studies on corn disease recognition, including the techniques used, data sources, disease categories and numbers, and the recognition accuracy of corn images.

**Table 12:** Performance comparison of relevant studies.

Techniques	Data sources	Categories and quantities	Acc (%)
SVM (Aravind et al., 2018)	PlantVillage	4 categories and 2000 images	83.7
SVM (Kusumo et al., 2018)	PlantVillage	4 categories and 3852 images	87.2
Improved LeNet (Ahila Priyadharshini et al., 2019)	PlantVillage	4 categories and 3852 images	97.89
DISE-NET (Yin et al., 2022)	Self-collected	5 categories and 1268 images	97.12
DMS-Robust Alexnet (Lv et al., 2020)	Self-collected	7 categories and 12227 images	98.62
CNN (Mishra et al., 2020)	Self-collected	3 categories and 4382 images	88.46
SKPSNet-50 (Zeng et al., 2022a)	Self-collected	6 categories and 1,452 images	92.9
LDSNet (Zeng et al., 2022b)	Self-collected	6 categories and 3363 images	95.4
DFCANet (Chen et al., 2022)	Self-collected	6 categories and 3271 images	98.47
Ours	Self-collected	6 categories and 3258 images	98.76

In these studies, CNNs showed significant performance strength, while conventional image processing techniques performed poorly in terms of recognition accuracy for multiple types of diseases (Aravind et al., 2018; Kusumo et al., 2018). Meanwhile, CNNs have shown better recognition performance in datasets with simple backgrounds (Ahila Priyadharshini et al., 2019; Lv et al., 2020; Yin et al., 2022), but the robustness of CNNs decreases when they are applied to corn disease identification in real environments (Zeng et al., 2022a). Moreover, our image dataset is similar to the studies of Chen et al. (2022) and Zeng et al. (2022b), but our accuracy is higher and the Flops is smaller. This indicates that FCA-EfficientNet has excellent computational efficiency and potential for practical implementation. Therefore, FCA- EfficientNet has certain performance advantages in the field of corn disease image recognition.

### 4.11 Deployed on Android

**Figure 17:**
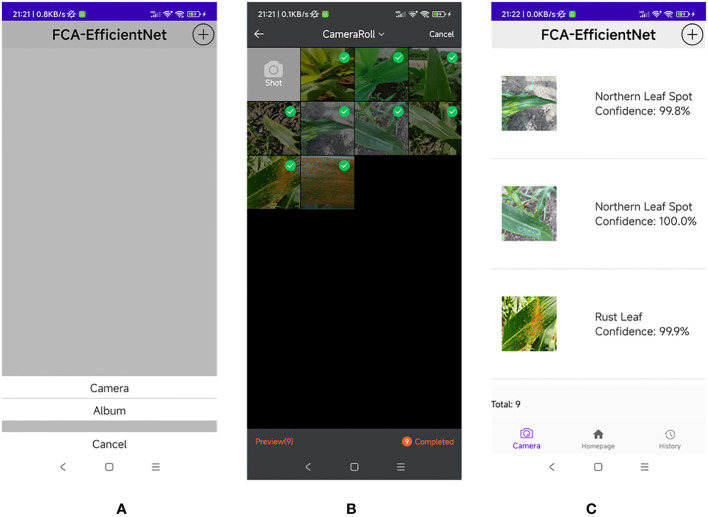
Application interface. **(A)** Open the application and select either “Camera” or “Album” to upload an image. **(B)** Open the photo gallery and select multiple disease images. **(C)** Obtain the disease image recognition results and their corresponding confidence levels.

We deploy the model on a mobile device to verify its performance. The mobile device used MediaTek Dimensity 1100, 8GB RAM, and Android 12. We conducted 5 consecutive experiments on this device, each experiment recognizing 1000 images. The average recognition speed for each image on the current device was: 88.34ms, 87.99ms, 91.87ms, 93.16ms, 93.02ms. The average recognition time for the 5 experiments was 92.88ms. Based on these current experimental results, the model can meet daily recognition needs. **Figure 17** shows the detailed operation of the system.

## 5 Conclusions

In this study, we propose an improved EfficientNet model, FCA-EfficientNet, to achieve accurate identification of multiple corn diseases. To recognize corn diseases in the field environment, we designed the AF module for filtering background noise and the FCA module that enables the network to be able to focus on diseased areas. In addition, we used LN and GELU to improve the generalization ability of the network. Finally, we removed redundant network structures to make the model more lightweight. In the corn disease dataset, the classification accuracy of FCA-EfficientNet for six corn images is 98.78%, and all the evaluation metrics in the comparison experiments are better than the classical CNN model with good generalization ability. The number of parameters of FCA-EfficientNet is 3.44M, which meets the conditions for deployment on mobile devices. The average recognition speed of each image on the test device is 92.88ms, which has good recognition speed and meets the recognition needs of growers. However, at the present time, the model can only quickly identify a small number of common corn diseases and cannot cover the full range of corn diseases. In the future, we plan to use transfer learning on large-scale plant disease datasets to enhance the model performance, as well as autonomously collect images of more types of corn diseases to further optimize the utility of the model and provide technical support to relevant growers.

## Data availability statement

The data analyzed in this study is subject to the following licenses/restrictions: The datasets for this manuscript are not publicly available because this research has been carried out as part of a project funded by the Government of China. Requests to access these datasets should be directed to xzhang1@gzu.edu.cn.

## Author contributions

XC and XZ designed the experiments. JC, RP, JWL, JML, LZ, and XW performed the experiments. JC, RP, and JWL analyzed data. JC drafted the manuscript. XC and XZ conducted visualization and proofreading of the manuscript. All authors contributed to the article and approved the submitted version.
